# Expert consensus on implementing patient-reported outcomes in telehealth: findings from an international Delphi study

**DOI:** 10.1186/s41687-025-00872-7

**Published:** 2025-04-09

**Authors:** Elizabeth J. Unni, Liv Marit Valen Schougaard, Olalekan Lee Aiyegbusi, Kedar K. V. Mate, Elizabeth J. Austin, Klara Greffin, Natasha Roberts, Birgith Engelst Grove, Holger Muehlan, ISOQOL Clinical practice SIG PROs in Telehealth Working Group

**Affiliations:** 1 Touro College of Pharmacy, New York, NY USA; 2 AmbuFlex, Centre for Patient-reported Outcomes, Gødstrup Hospital, Herning, Denmark; 3 Department of Clinical Medicine, Aarhus University, Aarhus, Denmark; 4 Centre for Patient Reported Outcomes Research (CPROR), Department of Applied Health Sciences, College of Medicine and Health, University of Birmingham, Birmingham, UK; 5 National Institute for Health and Care Research (NIHR) Birmingham Biomedical Research Centre, University of Birmingham, Birmingham, UK; 6 Faculty of Medicine, McGill University, Montreal, Canada; 7 University of Washington, Seattle, WA USA; 8 Department of Health and Prevention, Institute of Psychology, University of Greifswald, Greifswald, Germany; 9 School of Psychology, The University of Queensland, Brisbane, QLD Australia; 10 STARS Education and Research Alliance, Metro North Health, Herston, QLD Australia; 11 The University of Queensland, Herston, QLD Australia; 12 Division of Medical Psychology, Department of Medicine, Health & Medical University, Erfurt, Germany

**Keywords:** Patient reported outcomes, Patient reported outcome measures, Telehealth, Delphi, Expert consensus, Conceptualization, Applicability, Target population, Implementation challenges, Implementation strategies, Stakeholders

## Abstract

**Background:**

Using Patient Reported Outcomes (PROs) in clinical care can reduce healthcare service utilization by improving the quality of care. Telehealth, defined by WHO, as the use of “telecommunications and virtual technology to deliver healthcare outside of traditional healthcare facilities”, can facilitate a dynamic dialogue between patients and healthcare providers for timely interventions. With the increased use of telehealth facilitated by the infrastructure development during the COVID-19 pandemic, there is an opportunity to utilize telehealth for PRO implementation and a need for guidelines for using PROs via telehealth. This study aimed to generate expert consensus on the utilization of PROs in telehealth.

**Methods:**

Delphi methodology was used to achieve consensus among international experts with a predetermined consensus threshold of 70%. Experts were mainly identified through the ISOQOL Clinical Practice SIG. Surveys asked a combination of structured and open-ended questions about the conceptualization of PROs in telehealth, its applicability, target population, implementation challenges and successful strategies, evaluation approaches, and the essential stakeholders. Data from each round were iteratively analyzed using descriptive statistics (quantitative data) and content analysis (qualitative data).

**Results:**

Out of 24 invitations sent, 17 completed the first round, and 11 completed all three rounds. Respondents were equally distributed between clinicians and researchers and 70% had used PROs via telehealth before the pandemic. Consensus was achieved and some of the relevant aspects are monitoring patients for applicability; individuals with chronic diseases as the target population; resources, staff buy-in, and clinical workflow as the implementation challenges and strategies; utilization metrics for evaluation; and clinicians and patients as essential stakeholders. Though consensus was not reached for the conceptualization of PROs using telehealth, the modified FDA definition of telehealth with the addition of its purpose, and the mode of administration was the most acceptable version. See attached table.

**Conclusion:**

The expert consensus achieved provides important insights from an international perspective on how PROs are currently used via telehealth and the needed implementation support to advance their expansion in research and practice. Lack of consensus on the definition of PROs in telehealth signals the continued rapid evolution of their use and the need for additional research.

**Supplementary Information:**

The online version contains supplementary material available at 10.1186/s41687-025-00872-7.

## Background

The widespread adoption of digital technologies in healthcare has made the electronic delivery of healthcare services increasingly commonplace, with telehealth solutions playing a pivotal role. The COVID-19 pandemic further accelerated the adoption of digital technologies, especially in managing chronic disease conditions [1–3]. According to the World Health Organization (WHO), *telehealth* is defined as “the use of telecommunications and virtual technology to deliver healthcare outside of traditional healthcare facilities” [3]. Fundamental to most telehealth interventions is the integration of technology to facilitate a dynamic dialogue between patients and healthcare providers, relying on predefined data monitoring for timely interventions [4]. This approach extends to the management of chronic mental or physical conditions [5–9], offering advantages in symptom monitoring and health outcome assessment compared to traditional in-person healthcare delivery. For example, the utilization of wearable devices for remote vital sign tracking, such as blood pressure and glucose levels, enhances real-time data availability for healthcare professionals [10, 11]. Furthermore, virtual consultations enable timely health assessments, providing continuous care for individuals managing chronic conditions [12].

Telehealth solutions present significant advantages, including increased accessibility to healthcare services, reduced costs for patients and providers, and improved patient engagement through continuous remote monitoring [13]. They enable timely medical interventions, reduce the burden on healthcare facilities, and enhance convenience for individuals with mobility issues or those in remote areas. Additionally, telehealth fosters better chronic disease management by facilitating regular follow-ups and real-time health tracking [14, 15].

One strategy to maximize the potential of telehealth is the collection of *patient-reported outcomes* (PROs), defined as any report of a patient’s health condition directly from the patient without interpretation by a clinician or others [16]. With respect to the assessment of PROs in the context of telehealth in particular, it might be beneficial to distinguish between three different directives, even though these topics overlap to some degree: First, the provision of Patient-Reported Outcome Measures (PROMs) in telemedicine and digital health in general, are frequently used to evaluate the effectiveness of telemedical solutions such as to remotely monitor patient recovery, or to empower patients using a PROM-based feedback approach [17]. Second, more specifically, the emerging area of developing, validating, and implementing context-related PROMs, specifically devoted to telemedicine, are adopting a setting-sensitive approach to PROM assessment in telemedicine [18]. Third, the provision of PROs outside of traditional healthcare facilities through electronic formats (ePRO); for example, ePROs accessible via smartphones or tablets. Such ePROs offer novel possibilities when digitally and remotely collected, including real-time health status monitoring and flexible scheduling of hospital visits tailored to patient needs [19–20]. This approach has been successfully implemented across various chronic and malignant diseases [15]. Numerous randomized controlled trials have assessed the impact of PRO-based telehealth interventions in various chronic diseases, indicating comparable quality of care to traditional outpatient follow-up, with the potential to reduce healthcare service utilization [21–24]. Additionally, remote PRO monitoring in oncology care has shown positive effects on symptom burden, self-efficacy, health-related quality of life, and even survival [25–27]. Therefore, integrating PROs into telehealth has the potential to reshape follow-up activities for patients with chronic and malignant diseases.

However, alongside these benefits, it is essential to address the challenge of collecting valid and reliable PRO data. The effectiveness of telehealth solutions depends on the accuracy and integrity of the data collected, as this directly impacts clinical decision-making, diagnosis, and treatment outcomes. Variability in data collection methods, patient-reported inaccuracies, and differences in technological capabilities can introduce inconsistencies and compromise the quality of healthcare delivery [28]. The geographical distance between the patient and the healthcare provider may pose challenges in ensuring a valid assessment of the patient’s health status, particularly regarding symptoms like pain, burden of disease, or quality of life. While tools and guidelines related to PROs in clinical practice, such as the *International Society for Quality of Life Research (ISOQOL) User’s Guide for Implementing Patient-Reported Outcomes Assessment in Clinical Practice*, exist [29, 30], the distinctive features of PROs in telehealth demand specific attention. Factors such as remote completion of PRO assessments and the diverse range of patient populations with varying levels of technological literacy and device access necessitate the establishment of tailored guidelines and best practices. These guidelines play a crucial role in selecting suitable measures, ensuring data quality, upholding the integrity and confidentiality of patient-reported data, and facilitating the effective use and implementation of PROs in telehealth. With the increased use of telehealth facilitated by the infrastructure development during the pandemic, there is an opportunity to utilize telehealth for PRO implementation and thus there exists a need for guidelines for using PROs via telehealth. The overall aim of this study is to generate consensus from healthcare providers and researchers on the utilization of PROs in telehealth by (1) conceptualizing the scope of PROs in telehealth, (2) exploring the purpose and applicability of current PRO uptake in telehealth, and (3) identifying challenges and potential strategies for implementing PROs in telehealth.

## Methods

The study used the Delphi methodology to achieve consensus among experts in the use of telehealth for PRO collection [31]. Three rounds of online Delphi surveys were delivered through the University of Washington, Seattle Institute of Translational Health Science (IDHS) REDCap version 13.10.6. This study was deemed exempt by the Ethical Approval Board of the University of Washington as it involved professional opinions rather than personal or sensitive data, posed minimal risk to participants, and ensured that responses remained anonymous and aggregated. The predetermined threshold for consensus was set at 70%, in alignment with published guidance on Delphi studies [31, 32], which commonly define consensus thresholds between 70 and 80% depending on the research context. This threshold was chosen based on established methodologies in the literature, ensuring a balance between achieving meaningful agreement among experts while allowing for diverse perspectives. The research team (EU, LMVS, EJA, KM, HM, KG, BEG, NR, OLA) designed and conducted the study.

### Identification of potential participants

Experts were identified through the ISOQOL Clinical Practice Special Interest Group (CP SIG) and the personal networks of the research team. Invitations were sent to these individuals through the SIG or via e-mail and 24 individuals indicated their interest in participating in the Delphi rounds.

### Development and administration of the surveys

#### Delphi round 1 survey

The survey was developed by the research team based on a literature review and discussion. The survey had four domains which corresponded with the following themes – (1) conceptualization of PROs in telehealth, (2) applicability of PROs and the population in which it is used, (3) challenges of using PROs in telehealth and the successful strategies that have been used to overcome the challenges, and (4) recommendations for implementing and evaluating PROs in telehealth. The final survey comprised 16 questions which included ‘closed-ended’ questions with dichotomous answers (“yes” or “no”), multiple choice, and ranking options as well as open-ended questions that allowed participants to provide the required information in their own words.

The closed-ended questions were complemented by an “other” option. The closed-ended questions addressed (1) conceptualization of PROs in telehealth, (2) goals of the use of PROs in telehealth in their practice, (3) populations for which PROs in telehealth were used, (4) factors that were critical in choosing the optimal PROM in their practice, and (5) stakeholders needed for the successful implementation of PROs in telehealth in their practice. For the conceptualization question, respondents were provided with the FDA definition of PROs. Additional verbiage, “In the context of telehealth, the PROs can be administered via paper or electronic or verbal” was added and the participants were requested to indicate their agreement or disagreement with the proposed addition to the original FDA definition. If they did not agree, they were asked to add their definition in a free text box.

The open-ended questions asked about (1) three factors that help patients complete the PROs via telehealth, (2) three successful strategies that helped in implementing PROs via telehealth, (3) the challenges in implementing PROs via telehealth in technology, defining the purpose of using PROs, and competing priorities with clinical workflow, (4) other challenges faced while implementing PROs via telehealth, and (5) strategies used to evaluate the implementation success. Participants were also asked about their experience in using PROs via telehealth before the pandemic and if there was any change in the frequency of use of PROs via telehealth since the beginning of the pandemic. Demographic questions included primary clinical area of practice, role in the healthcare system, whether they have direct interaction with the patient, and how long they have used PROs via telehealth. Further details of the questions and their format can be found in the Supplementary file 1.

#### Delphi round 2 survey

The research team developed the Round 2 survey based on the results from Round 1. From the closed-ended questions, the feedback received on the conceptualization of PROs in the context of telehealth led to the proposal of three definitions for respondents to vote on. The closed-ended questions with ranking from the Round 1 survey were presented as percentages to obtain consensus. For the open-ended questions from Round 1, the respondents were asked to identify their top choices based on the qualitative coding results.

#### Delphi round 3 survey

The survey was further refined for Round 3 based on the results of Round 2. There were 6 close-ended questions in total. For the question on the conceptualization of PROs in the context of telehealth, respondents were asked to choose from 4 options which included the FDA definition of PROs, three new options, or suggest a new definition in a free text format. The remaining questions were dichotomous (yes or no) questions to facilitate the consensus process. Respondents who chose the ‘no’ option (did not agree with the Round 2 results) were presented with the ranking questions from Round 2 to complete.

### Data analysis

The study used both quantitative and qualitative methods to analyze the data. For the close-ended questions where the respondents were shown options and were asked to pick their top choices, descriptive analyses such as percentages were used. For those items that used open-text responses, the responses were qualitatively coded using directed content analysis [33] by a team of three trained qualitative researchers with expertise in PRO development and/or implementation (EJA, NR, KG) [34]. First, the coding team read through all open-text responses to familiarize themselves with the data. Next, the team double-coded all text responses in Microsoft Excel using a combination of deductive (guided by prior literature in PRO implementation) and inductive coding [35–38]. The coding team met iteratively to review, discuss, and reach a consensus on all coding. When possible, similar codes were grouped into a single, larger code. The team also made use of qualitative memos to document and align code applications across survey domains [39]. Finally, coding was reviewed with the full study team for further verification, interpretation, and consensus. Once consensus was reached on codes for all survey domains, the study team used results to guide the design of the second-round survey to Delphi participants.

## Results

Of the 24 experts who expressed interest in the study and to whom the survey was sent, 17 completed the survey, yielding a 71% response rate. Among the 17 participants, 16 granted consent for further contact in the Delphi rounds. Six individuals were lost to recruitment follow-up despite reminder emails. The experts’ experience in using PROs in telehealth ranged from two to eleven years. Twelve out of 17 participants reported the use of PROs in telehealth before the COVID-19 pandemic. Table 1 describes the characteristics of the study participants.

**Table 1:** Characteristics of expert survey participants

Ares of interests	• Oncology (*n =* 8) • Various clinical areas (*n =* 3) • Nonsurgical medicine (Rheumatology, Nephrology/Transplant; *n =* 2) • Orthopedics (*n =* 1) • Cardiology (*n =* 1) • Research (mostly in medicine: kidney & musculoskeletal disease, *n =* 1) • Did not specify their area (*n =* 1)
Role within the healthcare system	• Clinician and researcher (*n =* 7) • Researcher (*n =* 6) • Clinician (*n =* 1) • Technology administration (*n =* 1) • Other roles (*n =* 2)
Country	• Australia (*n* = 3) • Canada (*n* = 1) • Denmark (*n* = 2) • France (*n* = 2) • Germany (*n* = 1) • HongKong (*n* = 1) • Poland (*n* = 1) • United Kingdom (*n* = 2) • United Stated of America (*n* = 2)
Do you interact with patients?	• Yes (*n =* 9) • No (*n =* 8)

### Delphi round 1

#### Conceptualization

When queried about the conceptualization of PROs in telehealth, participants were presented with the U.S. Food and Drug Administration’s definition, stating that PROs are reports directly from patients about their health without clinician interpretation [16]. The research group added the sentence that in the context of telehealth, the PROs can be administered via paper, electronic, or verbal. Opinions on this conceptualization were diverse, with 12 participants in agreement, three in disagreement, and two choosing alternative responses. Participant comments (*n* = 5) elucidated concerns and suggestions. Notably, telehealth’s nature as an active patient-provider engagement medium may lead to the interpretation of PROs, and clinicians need the interpretation of (a change in) the PROMs to aid with their decision-making. Participants proposed refining the definition, especially distinguishing real-time (synchronous) from delayed (asynchronous) communication in telehealth. Questions arose regarding the interpretation of “verbal” in telehealth, specifying whether it involves synchronous communication via phone or computer. Further, emphasis was placed on the definition highlighting that PROs address subjective aspects of health reported exclusively by the patient. There were comments about the broad scope of “any report,” advocating for standardized reports using validated medical terminology adapted for patient understanding. In response to these comments, a suggested revision posited that “In the context of telehealth, PROs can be administered via paper, electronically, or verbally,” offering a more nuanced and adaptable framework for patient reporting for Round 2.

The codes that emerged from the qualitative analysis of the open-ended questions that included implementation challenges, successful strategies for implementation, factors that support PRO completion via telehealth, and evaluation approaches are shown in Table 2. These codes were incorporated into the Delphi Round 2 for consensus.

**Table 2:** Summary of qualitative coding results from the first-round survey for key domains

Domain	Salient codes identified & incorporated into Round 2
**Implementation challenges** **I**: *Defining the purpose* of PROs	· Lack of consensus across interdisciplinary teams · Difficulty satisfying the patient, provider, and organizational purpose of PROs · Low perceived value by providers and patients · Conflicting goals between research and clinical practice regarding the utility of PROs
**Implementation challenges** **II**: *Technology*	· Lack of organizational buy-in · Increased workload for existing clinical staff · Resources needed, including additional staff · Limited integration of PRO into the electronic medical record · Technology change requirement and management · Lack of vendor reliability (e.g., time it takes) · Limited user-friendliness of the telehealth platform for patients · Limited digital health literacy of patients · Limited reachability, especially for underserved patients (e.g., poor internet access) · Low use of the patient portal
**Implementation challenges III**: *Competing priorities*	· Acceptance issues (e.g., low perceptions of value or utility) · Workflow challenges · Lack of dedicated personnel and support · Lack of other dedicated resources and support (e.g., funding, organizational commitment) · High clinical workload · Increased respondent burden and time to complete PROs during the visit · Lack of infrastructure support unforeseen/unanticipated needs · Lack of patient engagement in PRO use · Limited and/or unclear guidance of PRO use (e.g., timing, clinical decisions)
**Successful implementation strategies**	· Staff and Management support (e.g., clinician champions, dedicated staff for implementation, staff training, endorsement from management, interprofessional agreement) · Staff and patient interaction (e.g., patient education, shared decision-making) · Patient inclusion (e.g., using PROs as part of care instead of research, patient engagement, patient reminders, patient preference) · Developing and/or optimizing clinical workflow (e.g., co-design of implementation and workflow with staff, minimal workflow interruptions, staff education, EMR integration) · Having adequate resources (e.g., staff, funding) · Ease of data collection (e.g., software usability, dedicated link to PRO) · Patient support for PRO completion (e.g., ease of use, clinical relevance) · Clear clinical application of PRO data (e.g., clinical use, patient follow-up, shared decision-making)
**Evaluation approaches**	· PRO utilization metrics · Measures of patient satisfaction · Measures of clinical satisfaction · Measures of clinical outcomes · Measures of healthcare service delivery outcomes · Patient engagement in care · Patient quality of life
**Factors that supported patients to complete PROs via telehealth**	• Usability • Accessibility • Clinical relevance to care • Encouragement and support from the healthcare team • Technology alignment with other IT systems

Table 3 demonstrates the analyses and steps taken in Delphi rounds 2 and 3.

### Delphi round 2

Based on the responses from the first round, a second survey was developed and administered to gain consensus from respondents. The second survey was sent to all 17 respondents from the first round. Fifteen experts responded to the survey (88% response rate) in a month’s time frame with reminder emails. The survey had both quantitative and qualitative items. When asking questions, the respondents were shown their responses from the previous round. As can be seen from Table 3, consensus was reached on 4 items. The items that needed to reach consensus were those that were qualitatively coded from Round 1.

### Delphi round 3

Based on the responses from the second round, a third survey was developed and administered to gain consensus on the six items. The third survey was sent to 15 respondents from the previous round. Eleven responded to the survey with a response rate of 73%. The survey was mainly quantitative to reach agreement on the themes developed by qualitative analysis coding from Round 2. It also had one question on the conceptualization of PROs in telehealth. In Round 3, consensus was reached on all items except the definition of PROs in telehealth. Even though the study did not reach a consensus on the definition of PROs in telehealth, the modified FDA version that states “PROs by telehealth, are defined as any report of the status of a patient’s health condition that comes directly from the patient, without interpretation of the patient’s response by a clinician or anyone else via telecommunication technologies, whether for routine clinical care or research purpose. When collected by telehealth, this can be administered via paper or electronic or verbal.” was the most acceptable version. Table 4 shows the consensus reached on all items from the study.

**Table 3:** Delphi survey methods for rounds 2 and 3

Item	Analysis from Round 1	What was shown to participants in Round 2?	What was asked in Round 2?	Results of Round 2	Results of Round 3
Definition of PROs in telehealth	Qualitative coding		Agree to the refined definition OR suggest alternative definition	Sent for Round 3 for consensus	Did not reach consensus
**Applicability of PROs**					
Major goals of using PROs at the practice	Quantitative	Showed all the 12 responses from previous round in decreasing frequency	Agree with the 5 most frequent responses OR indicate their top 5	Consensus reached	
Population in which the PROs are used	Quantitative	Showed all the 9 responses from previous round in decreasing frequency	Agree with the 5 most frequent responses OR indicate their top 5	Consensus reached	
**Challenges in implementing PROs in telehealth**					
Technology wise	Qualitative coding	Showed the nine themes from the coding	To select the top 5 themes	Participants selected the top 5 themes and was sent to Round 3 for consensus	Consensus reached
Defining the purpose of the PROs	Qualitative coding	Showed the four themes from the coding	To select the top 3 themes	Participants selected the top 3 themes and was sent to Round 3 for consensus	Consensus reached
Clinical workflow based on competing priorities	Qualitative coding	Showed the nine themes from the coding	To select the top 5 themes	Participants selected the top 5 themes and was sent to Round 3 for consensus	Consensus reached
Identify the factors that were critical in choosing the optimal PRO measure	Quantitative	Showed all the 10 responses from previous round in decreasing frequency	Agree with the 5 most frequent responses OR indicate their top 5	Consensus reached	
Successful strategies to implement PROs in telehealth	Qualitative coding	Showed the eight themes from the coding	To select the top 5 themes	Participants selected the top 5 themes and was sent to Round 3 for consensus	Consensus reached
Strategies used to evaluate the outcome of implementation success of the PROs in telehealth	Qualitative coding	Showed the seven themes from the coding	To select the top 5 themes	Participants selected the top 5 themes and was sent to Round 3 for consensus	Consensus reached
Stakeholders needed for the successful implementation of PROs in telehealth	Quantitative	Showed all the 8 responses from previous round in decreasing frequency	Agree with the 5 most frequent responses OR indicate their top 5	Consensus reached	

**Table 4:** Consensus reached on the research questions

Item	Consensus
Applicability of PROs - Major goals of using PROs at the practice	• Monitoring - adverse events/side effects/symptom monitoring • Monitoring - treatment/care outcomes or recovery • Patient Involvement in Care • Patient-Provider Communication • Quality Improvement
Applicability of PROs in Telehealth - Major population for which PROs are used	• People with chronic conditions • People who are recovering from illness/surgery • People who are older • People who are busy and miss appointments • People with mental health issues
Challenges faced while implementing PROs in telehealth - Technology	• Resources needed, including added staff • Organizational buy-in (e.g.: commitment) • Increased workload for existing clinical staff • Technology change requirement and management • Digital health literacy of patients
Challenges faced while implementing PROs in telehealth – Defining the purpose of using PROs	• Consensus for a shared purpose in an interdisciplinary team (e.g.: different stakeholders have different opinions) • Low value perception by providers and patients leading lack of buy in • Difficulty satisfying the patient, provider, and organizational purpose of the PROs • Conflicting goals between research and clinical practice regarding the utility of PROs
Challenges faced while implementing PROs in telehealth – Competing priorities with clinical workflows.	• Lack of dedicated personnel and support • Workflow (e.g.: clarification of whose responsibility, monitoring, time needed) • Clinical workload (e.g.: other projects, additional work) • Lack of patient engagement (e.g.: staff needed to engage patient in PRO completion) • Acceptance issues (e.g.: value, utility) • Guidance of PRO use (e.g.: timing, clinical decisions)
Implementing PROs in telehealth - Factors that were critical in choosing the optimal PRO measure for collection via telehealth.	• Ease of use (e.g.: length, response options, etc.) • Needed for clinical care decision making • Recommendations from guidelines • Measurement properties • Consensus between patients and clinicians
Successful strategies that worked in implementing PROs in telehealth.	• Staff and management support (e.g.: having a clinician champion, dedicated staff for implementation, staff training, endorsement from management, interprofessional agreement) • Developing a process for clinical workflow (e.g.: clinician buy-in, co-design of implementation and workflow with staff, minimal workflow interruptions, staff education, EMR integration) • Application of PRO data (e.g.: clinical use, for patient follow up, shared decision making) • Patient inclusion (e.g.: using PROs as part of care instead of research, patient engagement, patient reminders, patient preference) • Ease of data collection (e.g.: software used, user friendly, dedicated link to PRO)
Strategies used to evaluate the outcome of implementation success of the PROs in telehealth.	• PRO utilization metrics • Measure of clinical outcomes • Measure of health service outcomes • Measure of patient satisfaction • Measure of clinical satisfaction • Patient’s quality of life • Patient Engagement in their care
Top stakeholders needed for a successful implementation of PROs in telehealth	• Clinicians • Patients • Administration and support staff • Management • Information Technology Team

## Discussion

In this modified Delphi study, consensus was achieved on all the themes relating to the utilization of PROs using telehealth, except the conceptualization of PROs using telehealth. The majority of participants in the study agreed with the FDA definition of PROs, adding that in the context of telehealth, these measures can be administered by paper, electronically, or verbally and for routine clinical or research purposes. Though not a full consensus, the most accepted version was a modified FDA version, which stated “PROs by telehealth, are defined as any report of the status of a patient’s health condition that comes directly from the patient, without interpretation of the patient’s response by a clinician or anyone else via telecommunication technologies, whether for routine clinical care or research purpose. When collected by telehealth, this can be administered via paper or electronic or verbal”.

PROs, a standardized tool to develop shared clinical decision-making, can be further enhanced with the use of telecommunication technology for an active relationship between patients and providers. By consensus, the goals of PROs by telehealth were prioritized to include monitoring of symptoms and side effects, assessing treatment outcomes, engaging patients in care, improving communication, and evaluating care. These goals mirror the existing literature on using PROs in the clinical encounter [40]. Qualitative findings reported that PROs are particularly useful in oncology admission assessments for new patients. Applications for populations with chronic conditions were ranked highest, followed by those recovering from illness/surgery, those who are older, those who miss appointments, and those with mental health conditions. The populations identified are similar to those populations who benefit the best from telehealth [41].

A significant contribution of this study was the identification of challenges in implementing PROs by telehealth. The challenges were largely around the utility of PROs, integration with IT systems, and clinical workflows. It is worth noting that challenges also presented themselves when interdisciplinary teams in a clinic such as clinicians and researchers were not able to reach a consensus on the purpose of using PROs and organizational buy-in. The facilitators for the use of PROs in the literature are also reported to be context-specific [35], such as an onsite implementation support team for example. Consistent with our findings, the literature on PROs has also identified strategies for technological support, adequate resources, and organizational readiness as important [35]. Also consistent with the literature, structured evaluation using a wide range of outcomes was considered important by the experts in this study [35]. Barriers to both the clinical use of telehealth and the use of PROs highlight the potentially complex nature of implementation.

The broader implementation literature for the use of PROs in clinical practice supports our findings [35, 40–45], particularly on the importance of prioritizing the use of technology that aligns with other systems, and the importance of integration of PROs into clinical workflows [35–37]. The need for organizational readiness and adequate resourcing are reported to be important for the clinical implementation of both PROs and telehealth [46–47]. There is an emerging evidence base reporting the importance of evaluating implementation [48] and learnings from evaluation [35, 49] with our study identifying the importance of evaluation measures that assess patient, clinician and health service outcomes broadly. Stakeholders of importance were clinicians, patients, and administration staff, which also aligns with the available literature [50].

Research that investigates the experiences of patients in the use of PROs in clinical practice [51] and telehealth [52] identifies differing opinions between patients and clinicians on outcomes of importance. Similar findings have been found in studies investigating the experiences of patients and clinicians using PROs in telehealth [53–57]. For example, patients emphasized a lack of personal contact and their own ability to assess PRO data as barriers [54–55]. On the other hand, they pointed out that using PROs remotely enhanced flexibility and autonomy [54–56]. Clinicians revealed that using PROs in telehealth could make clinical assessments more mechanical and lack of the clinical gaze was a reported barrier [53, 57]. They also had concerns about the inclusion of patients, such as reflections on whether PROs in telehealth were only suitable for a selected group of patients [53].

The study also identified strategies such as usability, accessibility, clinical relevance, and engagement, with populations that may benefit including those that may struggle to engage with in-person appointments, such as those that do not attend scheduled appointments or those with mental health concerns. Though frameworks for both PROs and telehealth were in place before the COVID-19 pandemic, their uptake was accelerated with the pandemic crisis. Thomas and colleagues propose that this accelerated uptake can be sustained by (1) continuing to upskill workforces so that they are competent in telehealth, (2) supporting patients to be active users, (3) reforming funding models, (4) continuing to improve the “digital ecosystem”, and (5) making telehealth routine care [58]. There are calls to use the large amounts of data we have as a result of the COVID-19 pandemic [59] to evaluate how systems can be responsive and improve access to care for patient populations that may have previously struggled to engage.

A strength of our study is that the experience of our participants ranged from 2 to 11 years, they were drawn from an international audience in a range of settings, representing various professional backgrounds. In addition, with over 70%, the initial response rate to our invitations was high, which is a strength of the study as it indicates strong expert interest and engagement, enhances the representativeness of opinions, and contributes to the robustness of the findings. However, some limitations occur. Six participants dropped out between the Delphi rounds and most participants had experience in oncology, which may limit the generalizability of the findings to other medical fields, as discipline-specific perspectives could influence the results and may not fully represent the views of experts from other specialties. Additionally, though the study identified consensus on the purpose, goals, and applications of PROs in telehealth, no consensus was reached concerning the definition of PROs in telehealth. Future research could further explore this by conducting targeted qualitative studies to gain deeper insights into differing expert perspectives, engaging a broader range of stakeholders (e.g., patients, regulators, and technology developers), or utilizing Delphi rounds with more specific prompts to refine and align existing definitions.

In conclusion, this study highlights the varying opinions of experts, particularly clinicians, on the use of PROs via telehealth, such as the timing of PROs (synchronous versus asynchronous assessment) and the clinical relevance of the information collected.

## Electronic supplementary material

Below is the link to the electronic supplementary material.

Supplementary Material 1

## Data Availability

The datasets used and/or analysed during the current study are available from the corresponding author on reasonable request.
