# Supplementary material for: Expert consensus on implementing patient-reported outcomes in telehealth: findings from an international Delphi study
Source: J Patient Rep Outcomes. 2025 Apr 9;9:40. doi: 10.1186/s41687-025-00872-7 (PMC11981999; doi:10.1186/s41687-025-00872-7)
Supplement: Supplementary file 1 — Supplementary Material 1 [file 41687_2025_872_MOESM1_ESM.pdf]

Supplementary File 1: Delphi Round 1 Survey

1. Definition of PROs in Telehealth

Patient-Reported Outcomes (PROs) are defined as:
"Any report of the status of a patient's health condition that comes directly from the patient, without interpretation of the patient's response by a clinician or anyone else." (U.S. Food and Drug Administration, Guidance for Industry: Patient-Reported Outcome Measures, Dec 2009.)

In the context of telehealth, PROs can be administered via:

- Paper
- Electronic methods
- Verbal communication

Do you agree with this description of PROs in telehealth?

1. Yes
2. No
3. Other (please specify)

2. Major Goals for Using PROs in Telehealth

For using PROs via telehealth, what are the major goals at your practice? Please check up to FIVE reasons which are most important for which PROs are used in telehealth.

Goal Number	Goal
1	Monitoring - adverse events/side effects
2	Monitoring - treatment outcomes
3	Patient Satisfaction
4	Patient Education
5	Patient Reach
6	Patient Involvement in Care
7	Patient-Provider Communication
8	Quality Improvement
9	Reimbursement
10	Screening
11	Standard of Care
12	Treatment Evaluation
13	Please add any other reason:

3. Support

What were the top THREE factors that you think helped/supported patients to complete the PROs via telehealth at your practice?

4. Major Populations for PRO Use in Telehealth

When PROs are collected via telehealth, what is the major population for which it is used at your practice? Please check up to FIVE populations for which PROs are used in telehealth at your practice.

Number	Major
1	People who are older
2	Pediatric population
3	People who are busy and miss appointments
4	People with physical limitations or challenges
5	People with cognitive challenges
6	People with language challenges
7	People recovering from illness/surgery
8	People with chronic conditions
9	People with mental health issues
10	Please add any other populations

5. Successful Strategies

What were the top THREE successful strategies that worked in implementing PROs via telehealth at your practice?

6. Challenges in Implementing PROs via Telehealth

Implementing PROs via telehealth has challenges. Here are some of the common challenges. Please let us know whether each of this was a challenge for you at your practice and if so, why?

Number	Challenges
Technology<br>(+ text field)	1 = Yes, 0 = No
Defining the purpose of PRO use<br>(+ text field)	1 = Yes, 0 = No
Competing priorities with clinical workflows<br>(+ text field)	1 = Yes, 0 = No

7. Implementation

What were the THREE challenges faced at your practice while implementing PROs via telehealth?

8. Crucial Factors

When selecting PROs to be used via telehealth, which factors are critical in choosing the optimal PRO measure at your practice? Please check up to FIVE factors that are most crucial.

**Number**	**Factors**
1	Continued use of the same PROs as before the Telehealth implementation
2	Recommendations from guidelines
3	Conceptual Models / Theoretical Frameworks
4	Measurement Properties
5	Ease of Use – Length, Response options, etc.
6	Clinician Preferences
7	Patient Preference
8	Consensus between patients and clinicians
9	Needed for clinical care decision making
10	Needed for research purposes
11	Please add any other factors

9. COVID-19

Did you use PROs in telehealth before COVID-19?

1. Yes
2. No

10. Frequency

Has there been any change in the frequency of collection of PROs (either or not through telehealth) since the beginning of COVID-19 at your practice?

1. Increased
2. Decreased
3. Remained the same

11. Evaluation

What were the top THREE strategies used to evaluate the implementation success of the PROs in telehealth at your practice?

12. Key Stakeholders for PRO Implementation in Telehealth

Who were the top THREE stakeholders needed for the successful implementation of PROs in telehealth?

Number	Key Stakeholders
1	Patients
2	Clinicians
3	Information Technology Team
4	Department
5	Management
6	Administration and Support Staff
7	Payers
8	Researchers

13. Primary Clinical Area

What is your primary clinical area?

Number	Area
1	Many different areas / No specific area
2	Medicine (nonsurgical)
3	Surgery
4	Oncology
5	Orthopedics
6	Obstetrics
7	Pediatrics
8	Emergency Care
9	Intensive Care
10	Palliative Care
11	Psychiatry/Mental Health
12	Rehabilitation

13	Pharmacy
14	Laboratory
15	Radiology
16	Anesthesiology
17	Other

14. Department

Which specific department in Medicine?

15. Role in the Healthcare System

What is your role in the healthcare system?

Number	Role
1	Clinician
2	Clinician in training
3	Medical social worker / Rehabilitation
4	Researcher
5	Clinician/Researcher
6	Patient Partner
7	Administration/ Management
8	Technology Administration
9	Other

16. Duration

How long have you used PROs in telehealth?

Months (dropdown from 0-12):

Years (dropdown from 0-10 & more than 10):
